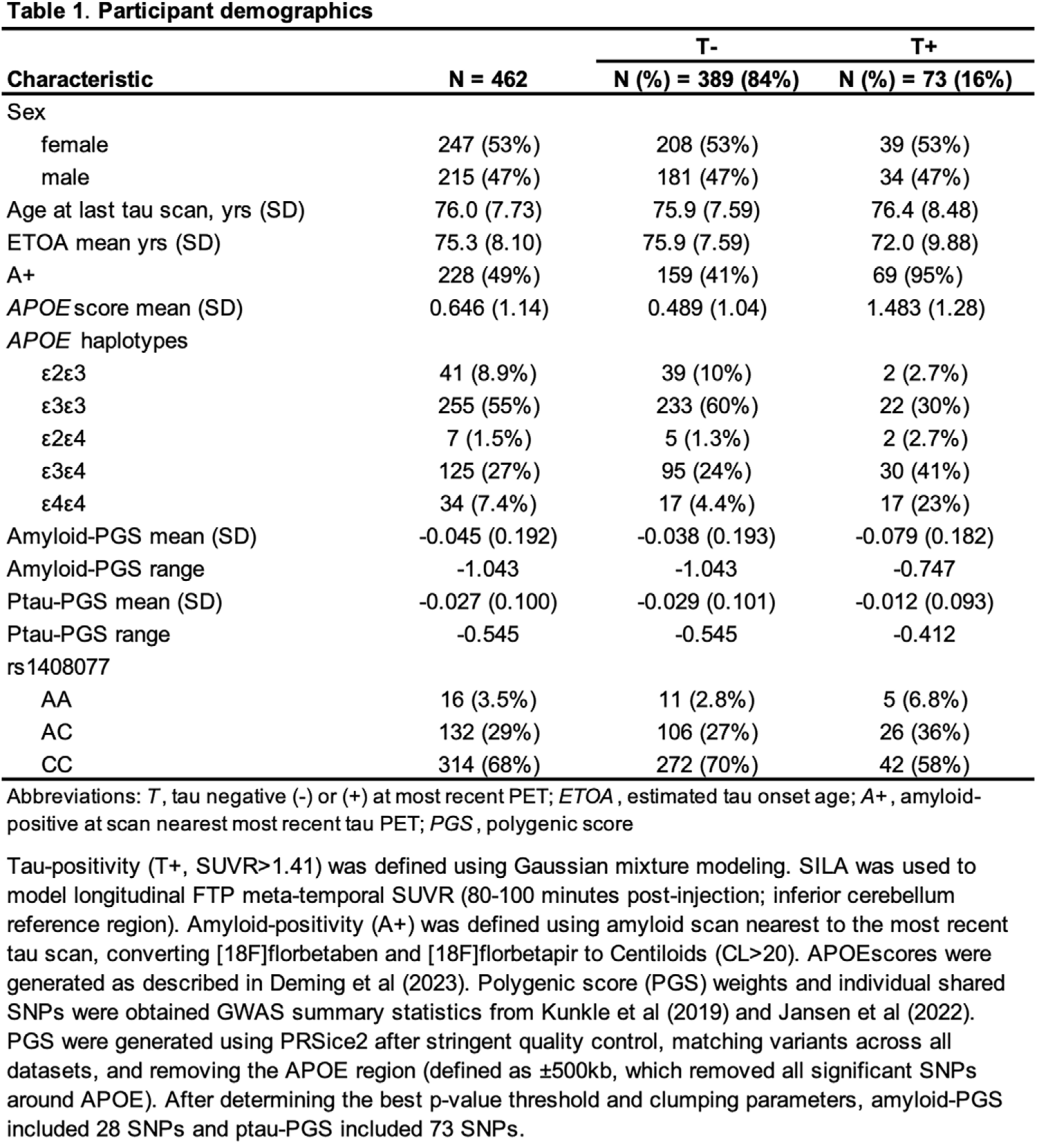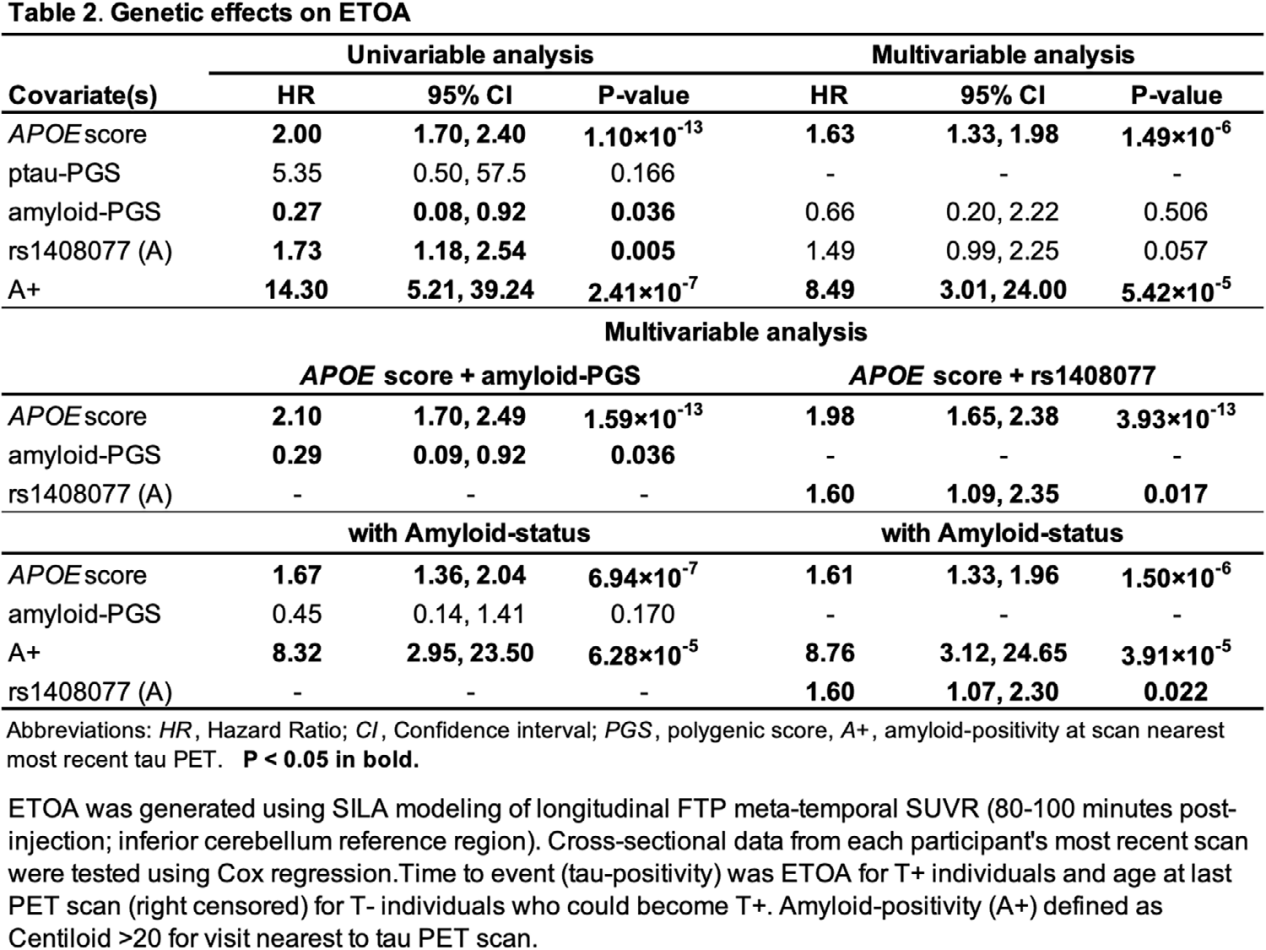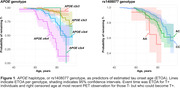# Alzheimer’s disease genetic risk factors' influence on tau onset age

**DOI:** 10.1002/alz.093671

**Published:** 2025-01-09

**Authors:** Yuetiva Deming, Jordan P Teague, Margo B. Heston, Yazan Hammad, Elena Ruiz De Chavez, Jacob Morse, Richard J Chappell, Rebecca E. Langhough, Megan L. Zuelsdorff, Corinne D. Engelman, Tobey J. Betthauser

**Affiliations:** ^1^ School of Medicine & Public Health, University of Wisconsin‐Madison, Madison, WI USA; ^2^ Department of Medicine, University of Wisconsin‐Madison School of Medicine and Public Health, Madison, WI USA; ^3^ Wisconsin Alzheimer’s Disease Research Center, University of Wisconsin School of Medicine and Public Health, Madison, WI USA; ^4^ University of Wisconsin ‐ Madison, Madison, WI USA; ^5^ University of Wisconsin‐Madison, School of Medicine and Public Health, Madison, WI USA

## Abstract

**Background:**

New methods developed to estimate when AD biomarkers became abnormal in individuals have shown considerable heterogeneity in amyloid and tau pathology onset age. This work used polygenic scores (PGS) generated from CSF Aß42 and ptau181 GWAS, individual‐level genetic data, and estimated tau onset age (ETOA) to identify genetic influences on tau onset beyond APOE.

**Method:**

Participants from the Alzheimer’s Disease Neuroimaging Initiative (ADNI) with genetic data, CSF biomarkers (Aß42 and ptau181), and longitudinal [18F]Flortaucipir (FTP) tau PET were analyzed (N = 462). Sampled iterative local approximation (SILA) was used to model longitudinal FTP meta‐temporal SUVR and generate ETOA for T+ and T‐ participants who could become T+, based on tau‐positivity (T+) defined using Gaussian mixture modeling (SUVR>1.41). Amyloid‐ and ptau‐PGS were generated from publicly available summary data, excluding the APOE region (±500kb). APOE neuropathology‐based scores (APOEscore) were used to test APOE. We analyzed individually a set of 14 SNPs that were previously associated with AD risk (P<1×10‐5) and both CSF biomarkers (P<0.05). Amyloid‐PGS, ptau‐PGS, APOEscore, and individual SNPs were tested as ETOA predictors using Cox regression modeling.

**Result:**

73 (16%) participants were T+ by their last tau PET (Table 1). APOEscore was significantly associated with earlier ETOA (P = 1.10×10‐13; Figure 1) as was the amyloid‐PGS (P = 0.036), but ptau‐PGS was not (P = 0.166; Table 2). An intronic variant in complement receptor 1 (CR1), rs1408077 (A allele, MAFEUR = 0.19) which was previously associated with higher AD risk and AD‐associated changes of CSF biomarkers, was also significantly associated with earlier ETOA (P = 0.005; Figure 1). The association remained significant (P = 0.017) after adding the APOEscore (P = 3.93×10‐13) and both were still significantly associated with ETOA (Prs1408077 = 0.022 and PAPOEscore = 1.50×10‐6) after accounting for amyloid‐positivity (P = 3.91×10‐5; Table 2).

**Conclusion:**

Our preliminary results support a key role for CR1 in the age when tau pathology becomes abnormal in AD. The tested variant, rs1408077, is in high linkage disequilibrium with a missense variant, rs2296160, and was previously associated with CR1 expression in the temporal cortex. This adds to previous studies that reported an interaction between CR1 and APOE‐e4 associated with episodic memory decline. Future work will include genome‐wide analyses of biomarker timing and multi‐trait analyses.